# Supplementary material for: RASCL: Rapid Assessment of Selection in CLades through molecular sequence analysis
Source: PLoS One. 2022 Nov 2;17(11):e0275623. doi: 10.1371/journal.pone.0275623 (PMC9629619; doi:10.1371/journal.pone.0275623)
Supplement: Attachment — Submitted filename: Response to Reviewers.docx [file pone.0275623.s018.docx]

September 14, 2022

Dr. Vladimir Makarenkov
Academic Editor
PLOS ONE

**RE: Response to reviewers for: RASCL: RAPID ASSESSMENT OF SELECTION IN CLADES THROUGH MOLECULAR SEQUENCE ANALYSIS.**

Thank you for the opportunity to submit a revised manuscript on “RASCL: RAPID ASSESSMENT OF SELECTION IN CLADES THROUGH MOLECULAR SEQUENCE ANALYSIS”.

Please find attached our revised contribution that incorporates responses to the Editor and Reviewer comments. Each of the comments have been addressed and a detailed response is attached. Both a marked-up manuscript and a clean LaTeX version of the paper are included.

We have taken special consideration to answer the important points raised by the reviewer regarding SARS-CoV-2 origins and genetic recombination detection, a limitation of our applications current implementation which we aim to handle in future versions of RASCL and that will enable it to play a more significant role in the global pathogen monitoring ecosystem.

Yours sincerely,

**Alexander G. Lucaci, M.S.**

Ph.D. Candidate in Bioinformatics
Institute for Genomics and Evolutionary Medicine (iGEM)
Temple University

**Comment #1**

I think that the authors should use recombination detection algorithms available for example in SimPlot++ (Samson et al., Bioinformatics 2022) to identify sequence change due to recombination.

**Answer #1** We have modified our manuscript to discuss the use and role of recombination detection in our application.

**Comment #2**
Also, I think that the paper could benefit from a short discussion about possible origins of SARS-Cov-2. You could rely on the following references in this discussion: Boni, Maciej F., et al. "Evolutionary origins of the SARS-CoV-2 sarbecovirus lineage responsible for the COVID-19 pandemic." Nature microbiology 5.11 (2020): 1408-1417. Domingo JL. What we know and what we need to know about the origin of SARS-CoV-2. Environ Res. 2021;200:111785. Makarenkov, V., Mazoure, B., Rabusseau, G. et al. Horizontal gene transfer and recombination analysis of SARS-CoV-2 genes helps discover its close relatives and shed light on its origin. BMC Ecol Evo 21, 5 (2021).

**Answer #2** We have modified our manuscript to include a discussion on the possible origins of SARS-CoV-2.
